# Recycled gold-reduced graphene oxide nanocomposite for efficient adsorption and photocatalytic degradation of crystal violet

**DOI:** 10.1038/s41598-024-54580-1

**Published:** 2024-02-22

**Authors:** Sherkawi H. Al-Ansari, Hassanien Gomaa, Rabeea D. Abdel-Rahim, Gomaa A. M. Ali, Adham M. Nagiub

**Affiliations:** 1 Department of Chemistry, Faculty of Science, Al-Azhar University, Assiut, 71524 Egypt; 2 Faculty of Science, Galala University, Suez, 43511 Egypt

**Keywords:** Reduced graphene oxide, Gold nanoparticles, Au@rGO nanocomposite, Electronic waste, CV dye, Adsorption, Photocatalytic degradation, Environmental sciences, Nanoscience and technology

## Abstract

In this study, gold-reduced graphene oxide (Au@rGO) nanocomposite has been synthesized by repurposing electronic waste and dry batteries. This innovative approach involved utilizing the graphite rod from dry batteries to produce reduced graphene oxide (rGO), which was subsequently modified through the incorporation of gold nanoparticles obtained from recycled electronic waste. This methodology marks a significant breakthrough in electronic waste recycling, presenting a cost-effective and sustainable means of creating novel nanocomposites for applications in photocatalysis and adsorption, particularly in the removal of crystal violet (CV) from aqueous media. The synthesized Au@rGO nanocomposite was characterized using X-ray diffraction, scanning electron microscopy, energy dispersed X-ray, and N_2_ adsorption/desorption. Parameters that affect the adsorption and photocatalytic degradation of CV dye have been studied in detail. The optimal conditions for CV adsorption and photocatalytic degradation were pH of 10, equilibrium time of 30 min, CV concentration of 10 mg/L and adsorbent dosage of 40 mg. Furthermore, the isotherm and kinetics of CV removal were also studied. The removal of CV dye using adsorption and photocatalytic degradation techniques reached 95% and 99%, respectively. Consequently, the results showed that photocatalytic degradation of CV dye onto the mesoporous Au@rGO nanocomposite is more proper way than the adsorption technique for removing the CV dye from aqueous media. The designed photocatalyst has high efficiency and it can be reused and activated several times so it can be used in real water treatment applications.

## Introduction

Recently, carbon material has gained significant attention from scientists and researchers due to its unique composition and characteristics^1–3^. One of the carbon compounds that has garnered particular interest is graphene, which is a single-layer graphite consisting of one-atom-thick hexagonal rings of SP^2^ hybridized carbon atoms^4^. Graphene sheets exhibit exceptional properties such as high thermal conductivity, electrical conductivity (5000 W/m K), and other interesting transport phenomena like the quantum Hall effect for each layer^5,6^. The remarkable features of graphene have led to numerous applications in various fields^7,8^. Graphene-based technologies have been employed in sensors, field emission devices, batteries, supercapacitors, membrane design, water desalination, and catalysis^9–11^.

One of main challenges in advanced nanocomposites manufacturing is the high cost. Therefore, finding low-cost precursors for these materials’ preparation is quite a promising approach from economic and environmental aspects^12,13^. Graphene could be obtained from zero-cost precursors such as graphite rod of the spent dry batteries. Furthermore, electronic waste (EW) accumulation has reached alarming levels, with approximately 25–40 million tons generated annually^14^. Improper disposal methods, including crushing, burying, or burning EW, have led to environmental pollution due to the release of hazardous components^15–18^. Innovative approaches are being developed to recycle EW and recover valuable components to combat this issue. Consequently, the global initiative to recycle EW, particularly computer parts like random access memory (RAM), aims to extract precious metals such as gold, silver, gallium, copper, platinum, tantalum, palladium, tellurium, germanium, and selenium^19–21^.

Among the various types of EW, computers, including laptops and desktops, contain essential hardware components such as a motherboard, central processing unit, RAM, hard drive, power supply unit, and expansion cards^22^. Given the significant annual production of computers globally, EW is expected to increase significantly.

Water purification has emerged as a critical humanitarian objective and has gained substantial research attention^23–26^. Industrial wastewater, containing various toxic organic compounds, has become a major environmental concern^27–30^. Industries such as leather, paper, plastics, printing, electroplating, and cosmetics contribute to the contamination of water sources with organic pollutants. Dye manufacturing produces a large number of toxic dyes like hetero polyaromatic, anthraquinone, and xanthinic dyes, which are difficult to remove from polluted water due to their high molecular weight^24,31–33^. These pollutants pose potential dangers to human health, including the risk of severe diseases like cancer. Therefore, effective separation of toxic dyes from industrial influent water is crucial before disposal into the environment. One commonly encountered cationic dye is crystal violet (CV), which is extensively used in the textiles, cosmetics, paper, printing, and leather industries^34^. Efforts have been made to decontaminate water through various methods such as filtration, electrochemical processes, precipitation, coagulation, and adsorption. Photocatalysis, an advanced oxidation process, has gained popularity in recent years due to its high efficiency, low cost, and absence of secondary toxic contaminants^35–40^. This process utilizes suitable catalysts and UV or sunlight to generate electrons and holes, which initiate oxidation–reduction reactions that degrade organic compounds on the catalyst's surface^41,42^. Photocatalytic degradation has shown promise as an eco-friendly and cost-effective method for treating wastewater, including the removal of CV dye^43–46^.

In this study, the mesoporous Au@rGO nanocomposite is prepared from waste precursors (graphite rod of spent dry battery and EW). In addition, the Au@rGO nanocomposite is used for the adsorption and photocatalytic degradation of CV dye in aqueous solutions.

## Experimental

### Chemicals

The chemicals used in this study were of a high purity grade and were not further purified. Sulfuric acid, orthophosphoric acid, nitric acid, hydrogen peroxide, potassium permanganate, glycerol, polyvinyl pyrrolidone (PVP), and CV were purchased from Sigma Aldrich Ltd. USA. Ethanol and hydrochloric acid were purchased from Merck, Germany. EW, RAM, and dry cell battery waste were collected from local shops.

### Characterization tools

The phase and structure of Au@rGO nanocomposite were verified using X-ray diffraction (XRD, Philips PW1700 diffractometer, Netherlands) with Cu-Kα radiation (λ = 1.541 Å). The scanning electron microscopy (SEM) analysis of the synthesized Au@rGO was conducted using a Carl Zeiss Sigma (500 VP and JSM5400 LV, Germany) to observe its surface morphology, equipped with energy dispersed X-ray (EDX). The surface area and porosity of Au@rGO were explored using N_2_ adsorption–desorption isotherms that was recorded using Quantachrom (Model Nova 3200, USA) at 77 K.

### Preparation of reduced graphene oxide from dry cell battery by modified Hummer method

Graphene oxide (GO) and reduced graphene oxide (rGO) were synthesized from graphite powder obtained from waste dry cell batteries. The graphite powder was first washed with water to remove impurities. The powder was then ground and crushed to obtain fine graphite powder. However, some inorganic materials remained in the powder, so it was treated with a mixture of hydrochloric acid and nitric acid (3:1) for 4 h. The mixture was then centrifuged and washed with distilled water until the pH reached neutrality. The recovered graphite powder was then dried at 60 ºC for 24 h. In a beaker, 270 mL of sulfuric acid was mixed with 30 mL of phosphoric acid (9:1), followed by stirring with 2.25 g of graphite powder. Then, 13.2 g of potassium permanganate was slowly added while stirring for 10 h until the color turned dark green. After that, 6.7 mL of hydrogen peroxide was added drop by drop over 10 min under stirring. The mixture was cooled for 30 min and then treated with 100 mL of hydrochloric acid and 300 mL of deionized water. Centrifugation was performed, and subsequent washes with distilled water were carried out. Finally, the sample was dried at 80 ºC for 24 h. To convert it into rGO, the dried sample was subjected to hydrothermal treatment at 150 ºC for 18 h^47–50^.

### Preparation of gold nanoparticles from electronic waste

**Figure 1:**
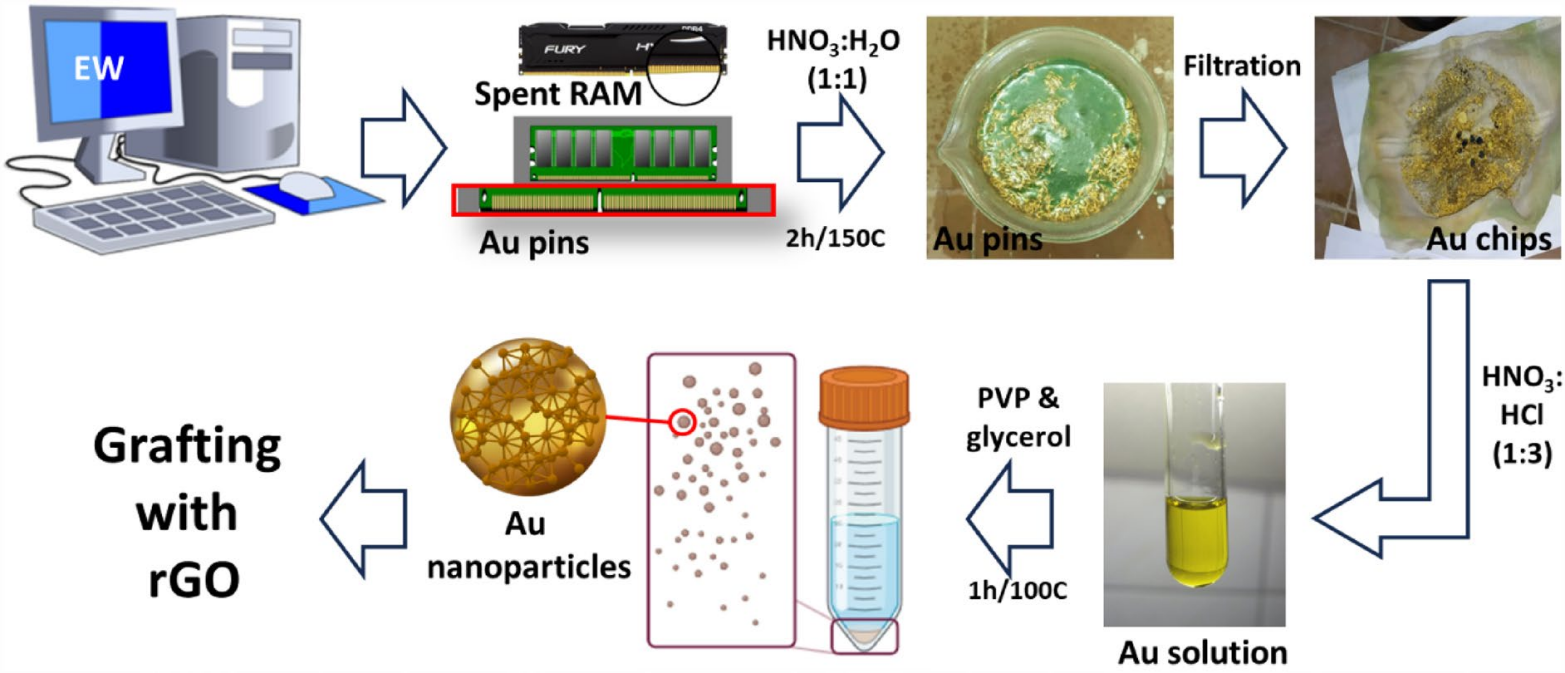
Schematic design of Au nanoparticles synthesis from EW.

The spent RAM was collected from EW collection stores, then pins were cut using scissors. Around 50 g of pins were added to a beaker containing 60 mL of nitric acid and 60 mL of deionized water (1:1) on a hot plate for 2 h at 150 ºC. This process facilitated the extraction of gold from the chips. Then, 0.18 g of Au chips was dissolved in 16 mL of aqua regia solution, which is a mixture of concentrated HNO_3_ and concentrated HCl in a ratio of 1:3. The gold solution was mixed with 0.115 g of PVP and 45 g of glycerol. The mixture was stirred for 1 h at 100 ºC, followed by centrifugation, washing, and then dispersed in 20 mL H_2_O to obtain gold nanoparticles suspension solution^51^ (Fig. 1).

### Preparation of Au@rGO nanocomposite

A solution of 20 mL of gold nanoparticles (9.5 mg/mL) was mixed with 0.44 g of rGO and stirred for 45 min. The mixture was then subjected to hydrothermal treatment at 150 ºC for 18 h. Afterwards, it was washed with deionized water and dried at 80 ºC for 24 h.

### Batch-adsorption and photocatalytic degradation of CV dye

Lab experiments were conducted to assess the adsorption capacity of materials for CV pollutants. Controlled conditions were maintained while conducting these experiments, where CV was adsorbed onto the materials. Furthermore, the efficiency of the Au@rGO for photocatalytic degradation of CV dye was observed using sunlight and examined extensively. A typical experiment was performed by adding 25 mg of Au@rGO photocatalyst to 25 mL with a concentration of 10 mg/L of CV dye. The mixture was then stirred magnetically at 70 rpm. Afterwards, the mixture of CV dye and the synthesized nanocomposite was rapidly centrifuged to eliminate the used Au@rGO. To ensure adsorption equilibrium between the CV molecules and Au@rGO, the mixture of CV Au@rGO was agitated in a dark room before exposure to sunlight for 30 min. A UV–Vis spectrophotometer was used to evaluate the supernatant resulting from centrifugation, which included the CV dye solution. The absorbance was measured using a quartz cuvette at a UV wavelength of 580 nm that was used as λ_max_. The removal efficiency (%) and the adsorption capacity (mg/g) of CV dye using the designed sorbent was calculated according to Eqs. 1 and 2, respectively^52^.

1 $$Removal\,\% = \frac{{\left( {A_{i} - A_{f} } \right)}}{{A_{i} }} \times 100$$

2 $$q_{e} = \left( {C_{i} - C_{f} } \right)\left( \frac{V}{m} \right)$$

To assess the reduction of CV dye from an aqueous solution by adsorption and photocatalytic degradation, measurements were taken for the initial and final CV absorbance (A_i_ and A_f_). Additionally, the initial and final CV concentrations (Ci and Cf) were determined. The volume of the CV solution (V) and the mass of Au@rGO (m) were also recorded. The intensity of incident sunlight was monitored at 30-min intervals using a Lutron LX-101 digital meter. The measured sunlight intensity ranged from approximately 185 × 10^2^ to 190 × 10^2^ lx. The experimental setup involved magnetic stirring of the mixture under direct sunlight between 11:00 AM and 3:00 PM on sunny days when the temperature was around 25 ± 2 °C. Various factors, including the quantity of adsorbent, duration, pH level, etc., were investigated to evaluate the efficacy of CV removal from the aqueous solution under different conditions. Subsequently, the optimal conditions for CV removal were determined.

## Results and discussion

### Au@rGO nanocomposite characterizations

#### X-ray powder diffraction

**Figure 2:**
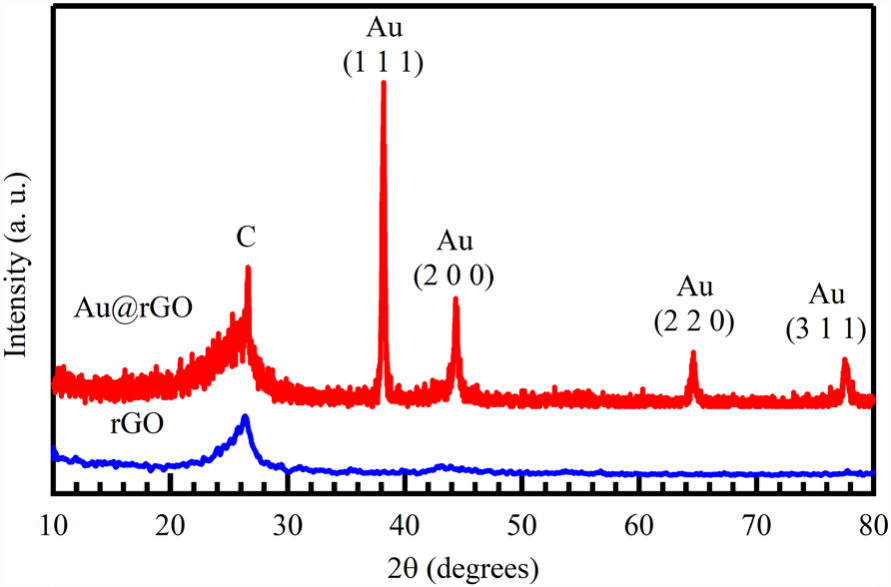
XRD patterns of the synthesized rGO and Au@rGO nanocomposite.

Figure 2 reveals that the XRD patterns of the rGO and Au@rGO nanocomposite are well-matched with the carbon and gold structure. In fact, no change was noticed in the previously prepared rGO structure^53^. The broad peak that characterize rGO at 25.81° is attributed to (002) plane 10. Diffraction peaks at 2θ° of 38.55°, 44.58°, 65.29° and 77.86° are assigned to the (111), (200), (220) and (311) diffraction planes, respectively, of the pure face-centered cubic (fcc) Au nanostructure. The obtained XRD data refer to the Au@rGO is synthesized successfully and Au particles are embedded in the rGO surface^54^.

#### Scanning electron microscopy and energy dispersed X-ray analyses

**Figure 3:**
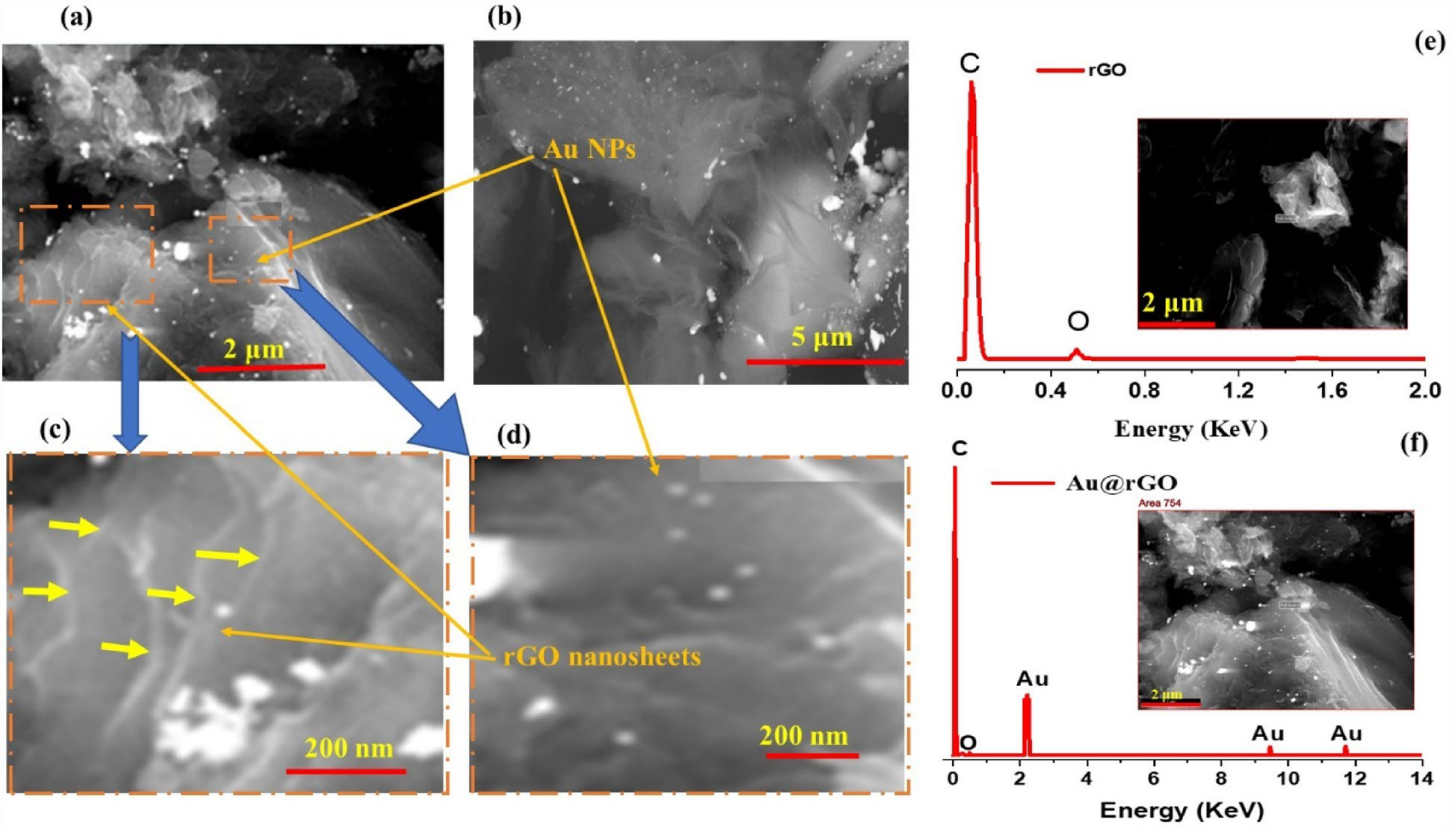
SEM images with different magnifications (**a**–**d**) Au@rGO nanocomposite and EDX analysis (**e** and **f**) for rGO and Au@rGO nanocomposite, respectively.

Due to its higher magnification, greater depth of focus, and ease of observing samples, the SEM was considered one of the most utilized devices in scientific research scope. Figure 3a–d demonstrates the SEM images of the synthesized Au@rGO nanocomposite at different magnifications. The figures clearly show the rGO layers had been synthesized successfully. Moreover, they demonstrate the gold nanoparticles and homogeneously distributed on the rGO surface.

Figure 3e,f displays the EDX spectra of the rGO and Au@rGO nanocomposite, respectively prepared by the hydrothermal method. The strong peak in EDX analysis at about 0.3 keV and small peak at around 0.5 keV are characterized by the carbon and oxygen, respectively, of rGO (Fig. 3e). While three peaks appeared in Fig. 3f, at about 2.23, 9.75 and 11.7 keV are attributed to the Au nanoparticles with weight ratio of 7.9%^55^. These results confirmed the formation of Au@rGO nanocomposite and Au nanoparticles were distributed on the rGO surface.

#### N_2_ adsorption/desorption study

**Figure 4:**
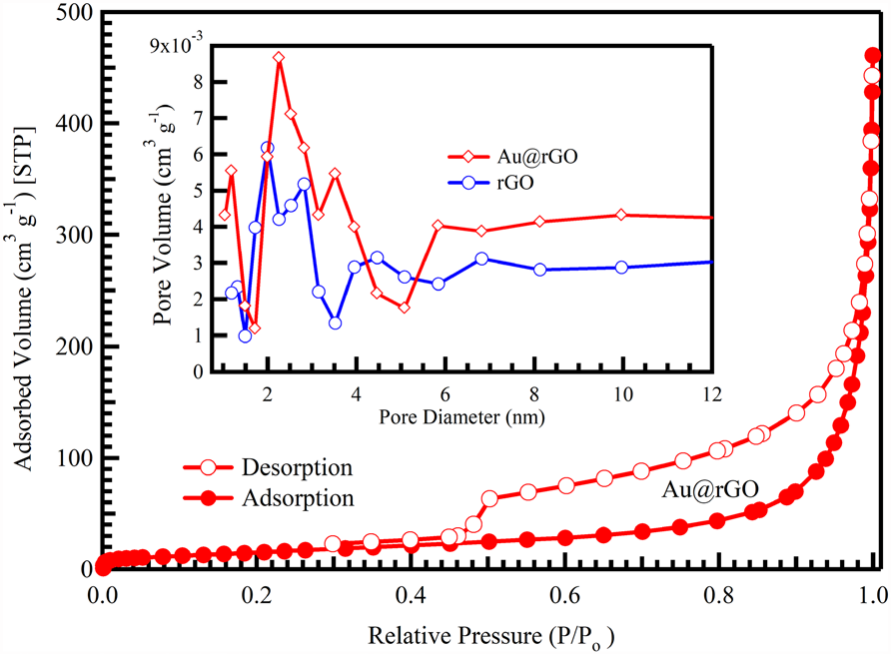
N_2_ adsorption/desorption and pore size distribution for rGO and Au@rGO nanocomposite.

The N_2_ adsorption/desorption isotherms Au@rGO are shown in Fig. 4 and the pore size distributions are displayed in the inset of the synthesized materials. The isotherm type was found to be type IV curves with hysteresis loops, demonstrating the mesoporous structure of the designed Au@rGO nanocomposites^56^. The pore size distributions for rGO and Au@rGO nanocomposite exhibit pore sizes ranging from 2 to 3 nm for rGO and from 2 to 4 nm for Au@rGO nanocomposite. It also shows that Au@rGO nanocomposite has a larger pore size and volume, which may be due to the appearance of more pores on the surface of the rGO. Consequently, these mesopores serve a vital function in increasing the adsorption efficiency during CV dye adsorption on the surface of Au@rGO sorbent. The specific surface areas of rGO and Au@rGO are 29.34 and 69.8 m^2^/g, respectively (calculated according to the details reported elsewhere^57^), and these data agree with the previous data that have been reported^10^.

### Optimization of adsorption and photocatalytic degradation processes

#### Effect of pH

**Figure 5:**
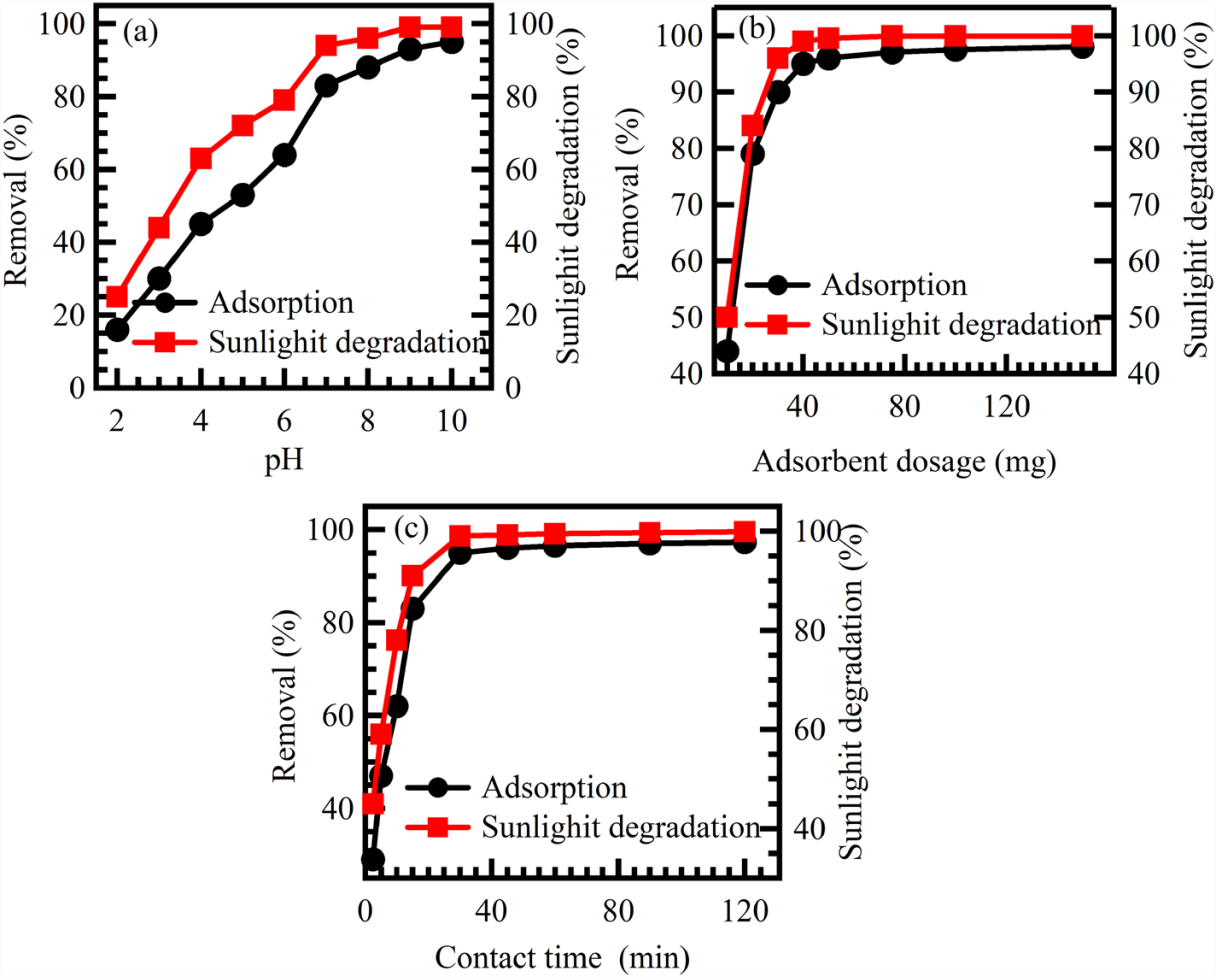
Effect of pH (**a**), dosage of adsorbent (**b**) and contact time (**c**) on the adsorption and photocatalytic degradation of CV using Au@rGO nanocomposite.

The pH of the solution has a significant impact on the surface charge of Au@rGO nanocomposite, which in turn affects the efficiency of CV adsorption and photocatalytic degradation. To investigate this effect, experiments were conducted at an adsorbent dose of 40 mg, CV concentration of 10 mg/L, and a solution volume of 40 mL under sunlight and room temperature. The results, shown in Fig. 5a, indicate that the efficiency of CV adsorption and photocatalytic degradation is limited to low pH values. This is because the surface of the Au@rGO nanocomposite is positively charged at low pH values, which reveals the positively charged CV molecules. As the pH of the solution increases, the surface of Au@rGO nanocomposite becomes more negatively charged, which attracts the positively charged CV molecules. This leads to an increase in the adsorption of CV molecules onto the surface of Au@rGO nanocomposite, hence, increasing its efficiency in the removal of CV from aqueous solutions. At alkaline pH values, CV removal efficiency using Au@rGO nanocomposite improves significantly. This is because the Au@rGO nanocomposite attracts CV molecules, which then undergo photocatalytic degradation under sunlight. At a pH of 10, the adsorption process exhibited removal of the CV dye efficiency of 95% approximately, while the photocatalytic degradation displayed removal efficiencies of about 99% for the CV. The improved performance observed at higher pH levels can be attributed to the presence of negative charges on the external surface-active sites of the Au@rGO nanocomposite. As CV is a cationic dye, it acquires a positive charge when dissolved in distilled water. Consequently, the interaction between the function groups with a positive charge of CV ions and the sites that have a negative charge of the Au@rGO nanocomposite adsorption catalyst becomes more pronounced at higher pH values. This enhanced interaction leads to greater rates of both adsorption and photocatalytic degradation of CV, resulting in higher removal efficiencies^58^.

#### Effect of adsorbent dose

Moreover, the amount of adsorbent also plays an important role in the efficiency of adsorption and photocatalytic destruction of various organic toxins^59–62^. It has a direct impact on the accessibility of surface-active sites for the entrapment of pollutants, thereby influencing the extent of color removal. To investigate this phenomenon, the effect of varying adsorbent doses ranging from 10 to 150 mg was examined on the adsorption and photocatalytic degradation of CV. Other variables such as solution volume, CV dye initial concentration, pH value of the solution, temperature, and contact duration were kept constant. The results in Fig. 5b demonstrated that increasing the quantity of adsorbent from 10 to 150 mg led to the increasing decolourization of CV contaminants. This enhancement can be attributed to the larger surface area and a greater number of active sites provided by the higher dosage of the adsorbent, resulting in improved entrapment of CV molecules. Therefore, an optimal adsorbent dose of 40 mg was determined for the removal of CV, exhibiting approximately 95% efficiency through adsorption and 99% efficiency through photocatalytic degradation^63^.

#### Effect of contact irradiation time

The contact irradiation time has a significant impact on the efficiency of CV adsorption and photocatalytic degradation on Au@rGO nanocomposites. This is because the contact irradiation time between the designed photocatalyst and CV dye affects the saturation state of the surface of the synthesized Au@rGO. As the contact time increases, the surface of the Au@rGO nanocomposite becomes more saturated with CV adsorbate, which leads to increased adsorption and photocatalytic degradation rates. The results shown in Fig. 5c demonstrate the influence of contact irradiation time on CV removal efficiency. The experiments were conducted by mixing 40 mg of adsorbent with 40 mL of 10 mg/L of CV dye solution under sunlight irradiation, 25 °C and a pH of 10. As contact irradiation time increased, both the adsorption and photocatalytic degradation of CV showed enhanced efficiency. Within 30 min, 95% of CV was removed through adsorption, while 99% was removed through photocatalytic degradation. Based on these findings, a contact irradiation time of 30 min was determined as the optimum condition for both methods^64^.

#### Effect of initial concentration on the dye adsorption and photocatalytic degradation

**Figure 6:**
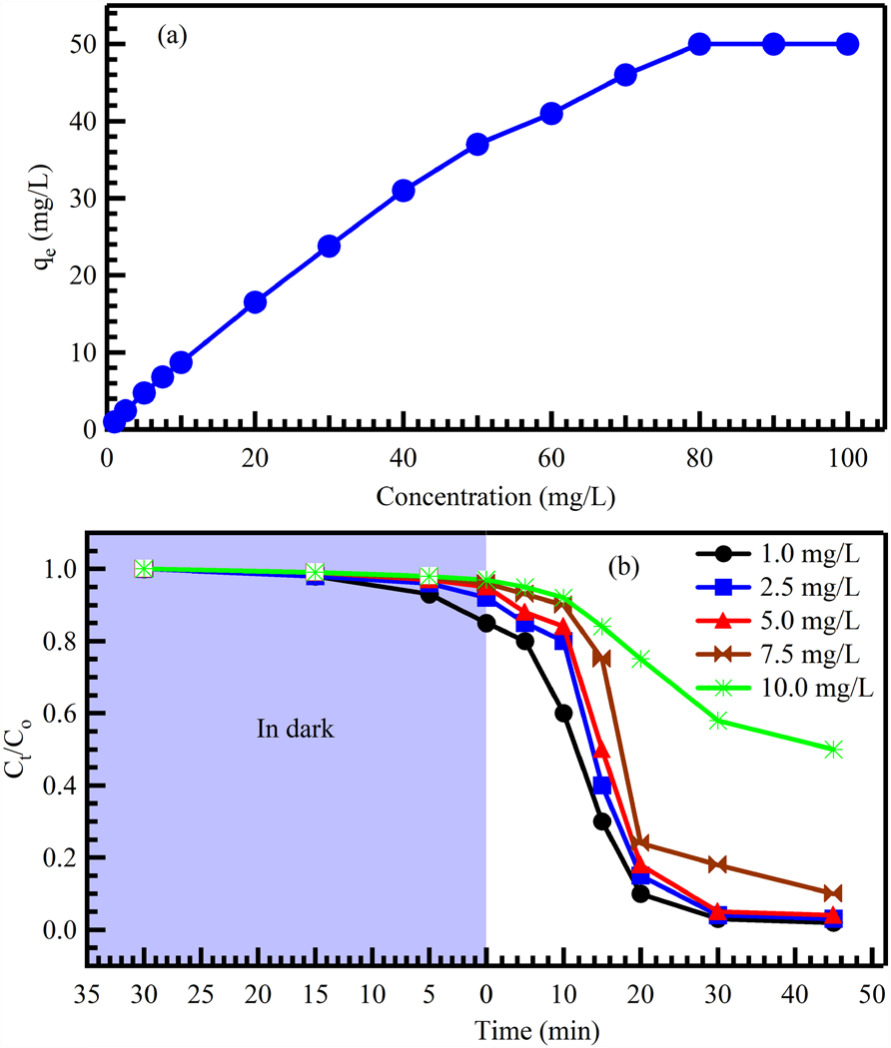
Influence of the concentration of CV dye on the adsorption capacity qe, (mg/g) (**a**) and time on photocatalytic degradation (**b**) CV dye using Au@rGO nanocomposites.

The effect of the initial concentration of CV was examined by varying the concentration from 1 to 100 mg/L, while keeping other factors constant, such as pH 10, adsorbent dose of 40 mg, and room temperature. The influence of the initial concentration of CV dye on its adsorption and photocatalytic degradation using Au@rGO nanocomposite was investigated, as depicted in Fig. 6. The adsorption process involved adding 40 mg of Au@rGO nanocomposite to 25 mL of CV solution with various concentrations of CV dye (ranging from 1 to 10 mg/L). The experimental conditions remained constant, with a pH value of 10 and a stirring rate of 70 rpm. Figure 6a illustrates that increasing the initial CV concentration enhances the adsorption capacity of CV dye on the Au@rGO nanocomposite until it reaches equilibrium (maximum adsorption). Based on the data obtained from Fig. 6a, the actual quantity of CV absorbed utilizing the Au@rGO adsorbent was approximately 50 mg/g. This can be attributed to the fact that higher CV concentrations result in shorter photon path lengths through the solution and coverage of active surface sites on the Au@rGO nanocomposite.

On the other hand, the catalytic photocatalytic degradation of CV dye was achieved by introducing 25 mg of Au@rGO nanocomposite into 25 mL of CV solution and stirring it at 70 rpm for 30 min in a closed dark reactor. The concentration of CV dye was adjusted as shown in Fig. 6b. The results indicate that increasing the CV concentration from 1 to 5 mg/L leads to a significant decrease in the effectiveness of CV dye degradation. This phenomenon can be attributed to the increased number of CV molecules occupying the surface-active sites of the synthesized Au@rGO nanocomposite, thereby reducing the diffusion and penetration of radiation to these sites^57,65^. As a result, there is a decrease in light absorption by the Au@rGO, leading to a reduction in the generation of electron–hole pairs and ultimately lowering the percentage of CV photocatalytic degradation^66^. Therefore, an optimized concentration for CV dye solution using a mesoporous Au@rGO nanocomposite is 10 mg/L or less^67^.

### Adsorption isotherms study

Investigation of the adsorption isotherm of CV dye on Au@rGO nanocomposite provides insights into the interactions between CV molecules and the adsorbent. To investigate the correlation between the equilibrium concentration (Ce) of CV and the adsorption capacity (qe) of Au@rGO nanocomposite, various concentrations of CV were employed under optimal adsorption conditions. Two commonly used adsorption isotherm models, Langmuir and Freundlich, were employed to understand the mechanism of CV adsorption. According to the Langmuir isotherm model, CV molecules adhere to the outer surface of Au@rGO nanocomposite, forming a monolayer through chemical interactions. This suggests that both the interior and active exterior sites of Au@rGO nanocomposite are involved in trapping CV molecules. The results suggest that CV molecules are adsorbed onto both the interior and active exterior sites of Au@rGO nanocomposite. The Freundlich adsorption isotherm model supports this, as it indicates that CV molecules form multiple layers on the surface of the nanocomposite through physical binding events. Overall, these findings provide valuable information about the type of interactions between CV dye and Au@rGO nanocomposite as an effective adsorbent. The research helps in understanding the mechanism of CV adsorption and can contribute to further advancements in designing efficient adsorbents for dye removal applications^68^. linear relations that describe Langmuir and Freundlich's isotherms were presented as follows:

3 $$\frac{Ce}{{{\text{q}}_{{\text{e}}} }} = \frac{1}{{{\text{K}}_{{\text{L}}} {\text{q}}_{{\text{m}}} }} + \left( {\frac{1}{{{\text{q}}_{{\text{m}}} }}} \right){\text{C}}_{{\text{e}}}$$

4 $$R_{L} = {\raise0.7ex\hbox{$1$} \!\mathord{\left/ {\vphantom {1 {\left( {1 + K_{L} C_{o} } \right)}}}\right.\kern-0pt} \!\lower0.7ex\hbox{${\left( {1 + K_{L} C_{o} } \right)}$}}$$

5 $$\ln \,{\text{q}}_{{\text{e}}} = \ln \,{\text{K}}_{{\text{f}}} + \frac{1}{{\text{n}}}\ln \,{\text{C}}_{{\text{e }}}$$

The theoretical CV adsorption capacity of Au@rGO nanocomposite is represented by Q_m_ (mg/g), while the constant of the CV adsorption equilibrium is denoted as K_L_. The proportional adsorption capacity of Au@rGO nanocomposite is indicated by K_F_, and the adsorption strength is represented by n. The value of n provides insight into the favorability of CV adsorption, where a higher value suggests stronger adsorption.

**Figure 7:**
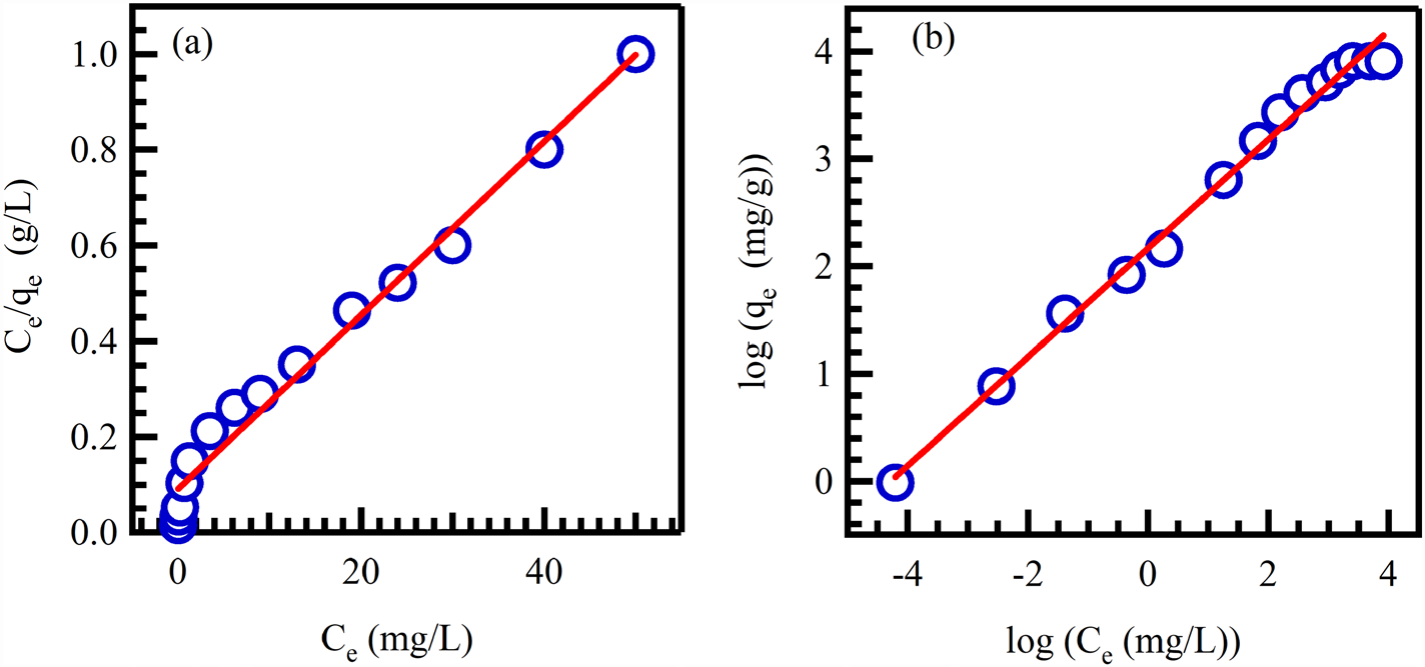
Langmuir (**a**), and Freundlich (**b**) isotherms for the adsorption of CV on Ag@rGO.

To estimate these constants, linear relations of Ce/q_e_ versus Ce and lnq_e_ versus lnC_e_ plots were used, and the slope and intercept of these relationships were determined. The R^2^ values indicate that Freundlich isotherm is a better fit compared to the Langmuir. In this case, the R^2^ value is 0.991, as shown in Fig. 7a,b and Table 1.

**Table 1:** Langmuir and Freundlich isotherm parameters of CV adsorption.

Langmuir	R^2^	q_m_ (mg/g)	K_L_ (L/mg)	R_L_
	0.979	55.9	0.189	0.642
Freundlich	R^2^	n	K_F_ (mg^1−(1/n1/n^/^g^)	1/n
	0.990	1.974	8.6711	0.5065

Based on the data of the Langmuir isotherm model, the maximum CV adsorption capacity on the surface of Au@rGO nanocomposite was determined to be 55.9 mg/g. Furthermore, the reversibility factor (R_L_) for the adsorption of CV dye on the surface of Au@rGO nanocomposite was determined to be 0.642. This is less than 1, which indicates that the adsorption process is favorable and fully reversible. In other words, the Au@rGO nanocomposite adsorbent can effectively release the trapped CV molecules using a suitable eluent agent. Table 2 compares the main adsorption findings with some related materials with their main characteristics and experimental conditions.

**Table 2:** Comparison of CV adsorption capacities using some related adsorbents.

Adsorbents	Removal capacity (mg/g)	Removal efficiency (%)	Optimum adsorption conditions	Ref
Au@rGO	55.9	99	The initial concentration of CV dye is 10 mg/L, pH = 10, the adsorbent dosage is 40 mg, T = 298 K and the equilibrium time is 30 min	This work
GO	32.12	94.86	The initial concentration of CV dye is 50 mg/l, with 0.1 g/L adsorbent dosage and 200 mg/L adsorbate content *at T* = 303 K and the contact time is 60 min	^71^
Graphite	33.48	93.25		
Graphene quantum dot	34.46	99.10		
Sugarcane bagasse and calcium oxide with ammonium hydrogen carbonate composite	293.02	97.67	The adsorbent dose is 3.33 mg/mL, pH 9.0, at T = 313 K and initial dye concentration 1000 mg/L and t_eq_ = 80 min	^72^
Phosphorus-doped carbon composite	1123	97	The initial concentration of CV and MB dyes are 1200 and 914 g/l, respectively, pH = 8, T = 298 and t_eq_ = 24 h	^73^
Pumice	6.99	86.68	initial dye concentration of 100 mg/L, pH of 6.5, T = 298 K, contact time of 150 min, adsorbent dosage of 0.5 g and teq = 150 and 120 min. for MG and CV, respectively	^74^
Montmorillonite-filled sodium alginate/gelatin	1000	92.1	The initial concentration of CV dye is 20 mg/L, adsorbent dose. Is 0.025 g/50 mL, pH = 7, a temperature of 25 °C and t_eq_ = 120 min	^75^
Binary g-4/ZnV_2_O_4_ nanocomposite	384.61	99.67	Initial concentration of CV dye is 20.048 ppm, adsorbent dosage of 19.776 mg, contact time of 59.20 min, and pH of 8 and t_eq_ = 59.2 min	^76^
Modified rice husk	90.02	96.16	Initial concentration of CV dye is 50 mg/L, adsorbent dose of 0.025 g, agitation speed of 190 rpm, T = 298 K, pH = 10 and t_eq_ = 70 min	^77^
Multiwalled carbon nanotubes	228.3	96	Initial concentration for both dyes is 15 mg/L, T = 298 k, adsorbent dos = 10 mg/30 mL and teq = 120 min	^78^

The Freundlich isotherm was used to analyze the adsorption data. The value of n was measured to be 1.974, suggesting that the CV adsorption process is a reasonable or hard adsorption method. Additionally, the value of 1/n was found to be less than 1, implying that the adsorption of CV dye on Au@rGO is favorable and involves a chemisorption process. The presence of microporosity in the Au@rGO nanocomposite facilitates the diffusion of CV molecules within the adsorbent. This makes it a promising option for the removal of CV contaminants.

### Photocatalytic degradation mechanism

**Figure 8:**
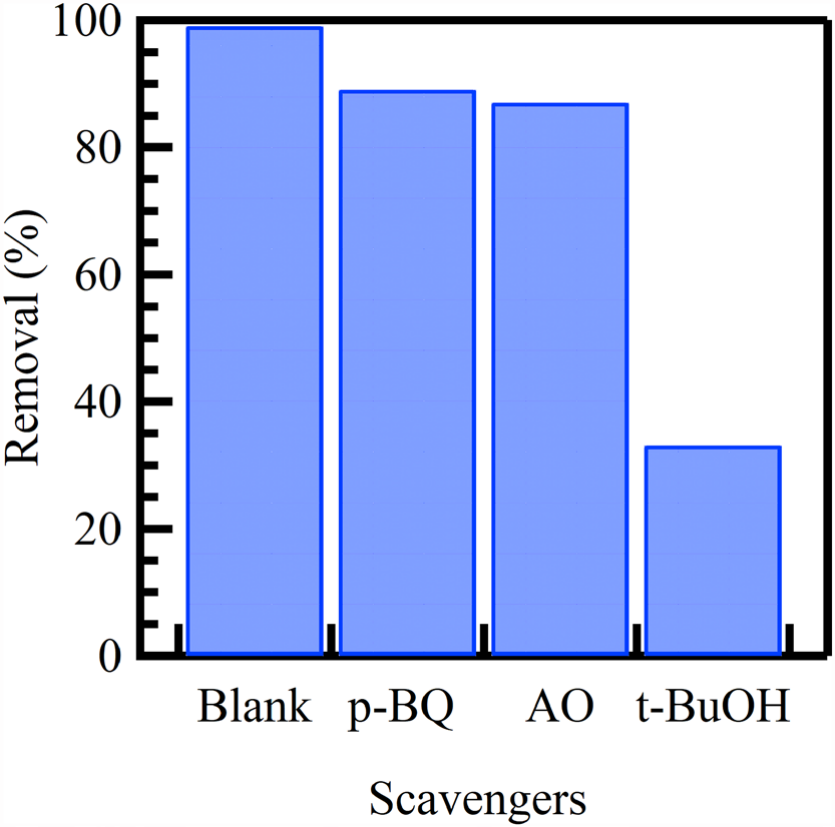
Impact of various scavengers on the photocatalytic degradation of CV dye using Au@rGO nanocomposite.

To determine the main active species governing the degradation of CV dye using Au@rGO nanocomposites, a series of experiments were conducted employing various scavengers, including p-benzoquinone (p-BQ) to quench O_2_^−·^ radicals, ammonium oxalate (AO) to scavenge holes, and tert-butyl alcohol (t-BuOH) to capture OH^·^ radicals. These scavengers were introduced into the CV solution before UV irradiation under the optimized removal conditions. As illustrated in Fig. 8, adding AO and p-BQ exhibited negligible effects on the CV degradation performance, suggesting that holes (h^+^) and O_2_^−·^ radicals have an inconsequential impact on the CV degradation. Conversely, the inclusion of t-BuOH distinctly diminished the CV degradation rate. Thus, the preeminent active species in the photocatalytic degradation of the CV pollutant are identified as OH^·^ radicals.

**Figure 9:**
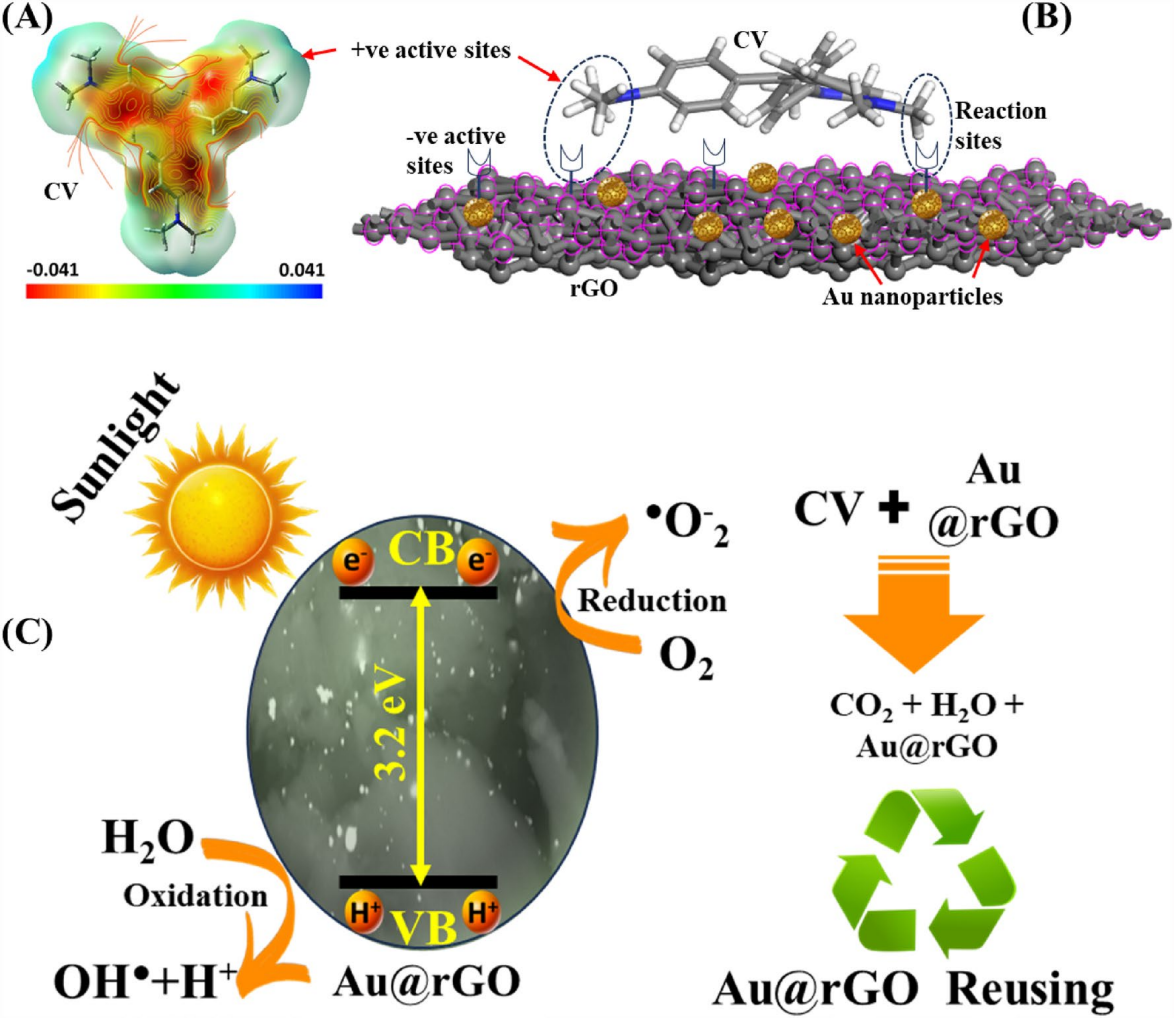
Representation of the molecular electrostatic potential of CV dye (**A**); Representation the reaction sites of CV dye onto Au@rGO nanocomposite surface (**B**); Schematic design of CV degradation using Au@rGO nanocomposite (**C**).

This finding suggests that the mechanism of CV adsorption onto Au@rGO nanocomposite involves layer formation through chemical bonds, as shown in Fig. 9.

**Figure 10:**
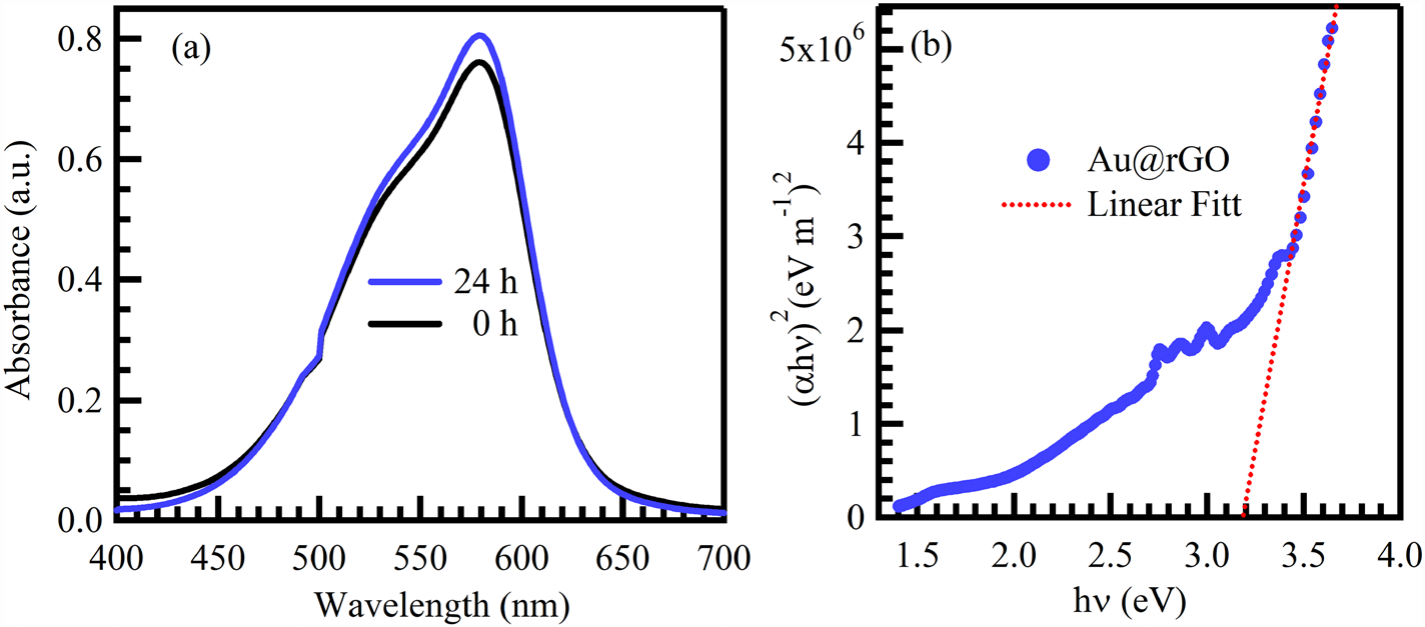
Direct photolysis of CV dye under sunlight and without using photocatalyst (**a**), and Tauc plot (**b**) of Au@rGO nanocomposite.

The experiment under solar irradiation was carried out using 25 mL of 10 mg/L CV solutions. The absorbance of CV dye before and after 24 h was recorded using UV–Vis spectrophotometer. Based on the results in Fig. 10a, more than 98% of CV remained after 24 h in the direct photolysis test.

The photocatalytic degradation of CV dye using Au@rGO nanocomposite under UV light excitation entails the generation of electron (e^−^) and positive electron–hole (h^+^) pairs, a consequence of the excitation of an electron (e^−^) from the valence band (VB) to the conduction band (CB). The determination of the bandgap, achieved through the Tauc method utilizing UV–vis spectra of Au@rGO nanocomposite, is encapsulated in the subsequent equation^69^:

6 $$\left( {\alpha h\nu } \right) = A \left( {h\upsilon - E_{g} } \right)^{2}$$

where E_g_ represents the bandgap of Au@rGO nanocomposites, h denotes Planck's constant, υ signifies frequency, α corresponds to the absorption coefficient, and A is the proportionality constant. As illustrated in Fig. 10b, the bandgap computed for Au@rGO nanocomposite is approximately 3.2 eV. Under UV light, H_2_O molecules can engage with holes (h^+^_VB_), generating OH^·^, while e^−^_CB_ can capture O_2_ molecules, producing anionic superoxide (O_2_^−·^) radicals at the Au@rGO surface. The ensuing reaction between O_2_^−·^ and H^+^ ions yield hydroperoxy (HOO^·^) radicals. Subsequently, the HOO^·^ radical undergoes further transformation into H_2_O_2_ and O_2_. The dissociation of H_2_O_2_ yields additional OH^·^ radicals, which play a pivotal role in the degradation of CV dye, as exemplified in the subsequent equations^70^:

7 $${\text{Au@rGO}} + {\text{h}}\upupsilon \to (e_{CB}^{ - } + h_{VB}^{ + } ){\text{@Au@rGO}}$$

8 $$H_{2} O + h_{VB}^{ + } \to OH^{.} + H^{ + }$$

9 $$O_{2} + e_{CB}^{ - } \to O_{2}^{ - .}$$

10 $$O_{2}^{ - .} + H^{ + } \to HOO^{.}$$

11 $$2HOO^{.} \to H_{2} O_{2} + O_{2}$$

12 $$H_{2} O_{2} \to 2OH^{.}$$

13 $$CV + OH^{.} \to degradation\,products$$

### Adsorption kinetics study

To discover the CV dye removal process and gain insight into its mechanism under ideal adsorption conditions, two frequently utilized kinetic models, the pseudo-first-order (PFO) and pseudo-second-order (PSO) models, were utilized to analyze the experimental findings^79^. The adsorption kinetics were examined over 2.5 to 30 min. PFO and PSO models can be calculated according to the following equations^80^:

14 $$log\left( {q_{e} - q_{t} } \right) = log\,q_{e} - \left( {\frac{{k_{1} }}{2.303}} \right)t$$

15 $$\frac{t}{{q_{t} }} = \frac{1}{{k_{2} q_{e}^{2} }} + \left( {\frac{1}{{q_{e} }}} \right)t$$

**Figure 11:**
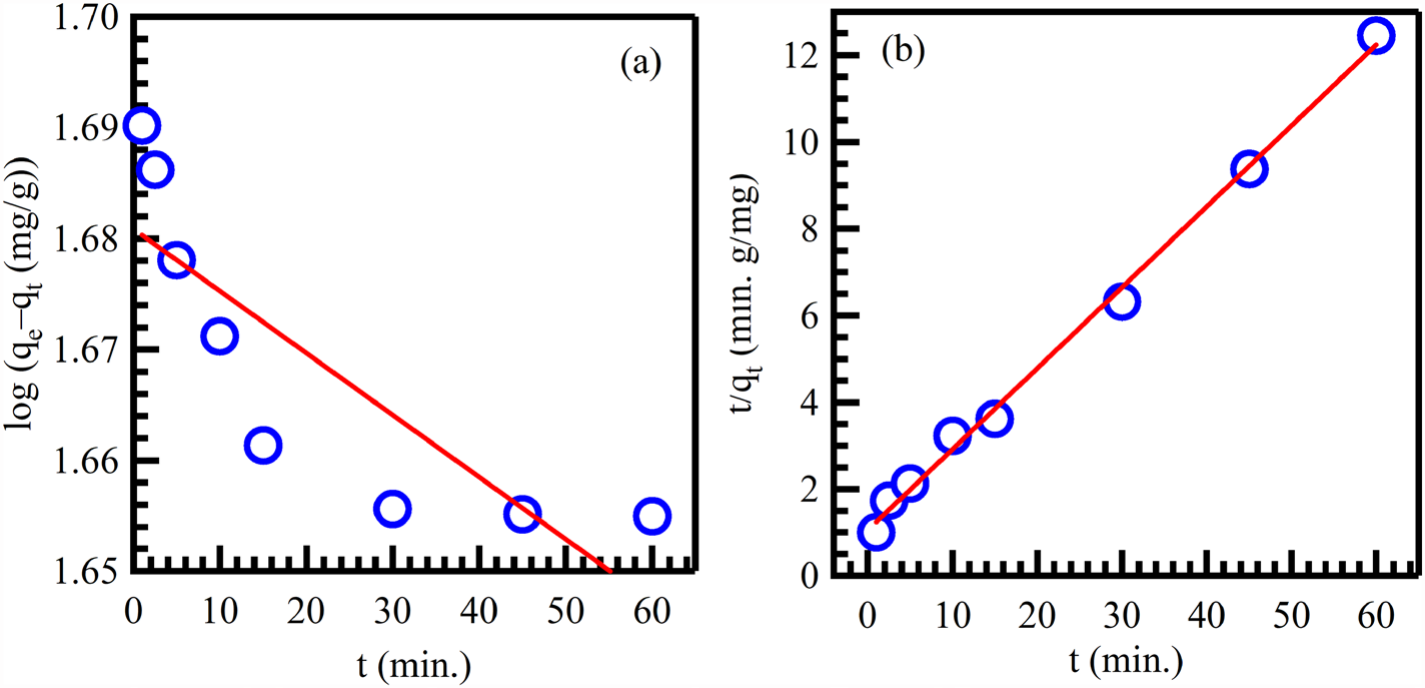
Pseudo-first-order (**a**), and pseudo-second-order (**b**) kinetics for adsorption of CV onto Au@rGO.

In these equations, k_1_ and k_2_ represent the adsorption rate constants for PFO and PSO, respectively. Q_e_ indicates the amount of adsorbed CV dye in mg/g of Au@rGO nanocomposite adsorbent at equilibrium while q_e_ indicates the amount of adsorbed CV dye by mg/g on the sorbent at time t. To determine the parameters and constants, linear plots of Log(q_e_-q_t_) and t/q_t_ versus time were analyzed, as shown in Table 3 (Fig. 11a,b). The R^2^ values of the fitted lines indicated that the adsorption of CV dye adhered more closely to the PSO than to the PFO model. This suggests that the removal mechanism of CV involves chemical adsorption through the utilization of Au@rGO nanocomposite. During the adsorption process, the decolourization of CV was initially rapid but slowed down as it approached equilibrium. The PSO model provided an estimated q_e_ value that was very close to the experimental q_e_ value when the initial concentration of CV dye was 25 mg/L.

**Table 3:** Kinetic parameters of PFO and PSO for the CV dye adsorption and photocatalytic degradation.

Adsorption	Pseudo-first-order			Pseudo-second-order			Langmuir–Hinshelwood		
	R^2^	K_1_ (min^−1^)	q_e (_mg/g)	R^2^	K_2_ (g/mg.min)	q_e_ (mg/g)			
	0.665	12.872	47.86	0.996	0.420	5.387			
Photocatalytic degradation	R^2^	K_1_	t_0.5_	R^2^	K_2_	t_0.5_	R^2^	K_r_ (mg/L.min)	K_s_ (L/mg)
	0.9981	0.0676	7.96	0.9850	0.5534	0.328	0.9473	12.87	0.459

### Photocatalytic degradation kinetic study

The PFO and PSO linear model equations were utilized to analyze the kinetic rate of CV-photocatalytic degradation with the Au@rGO. The equations for the PFO and PSO kinetics models of photocatalytic degradation are as follows^79,81,82^:

16 $$ln\frac{{C_{o} }}{{C_{t} }} = k_{1} \times t$$

17 $$\frac{1}{{C_{t} }} = \frac{1}{{C_{o} }} + k_{2} \times t$$

**Figure 12:**
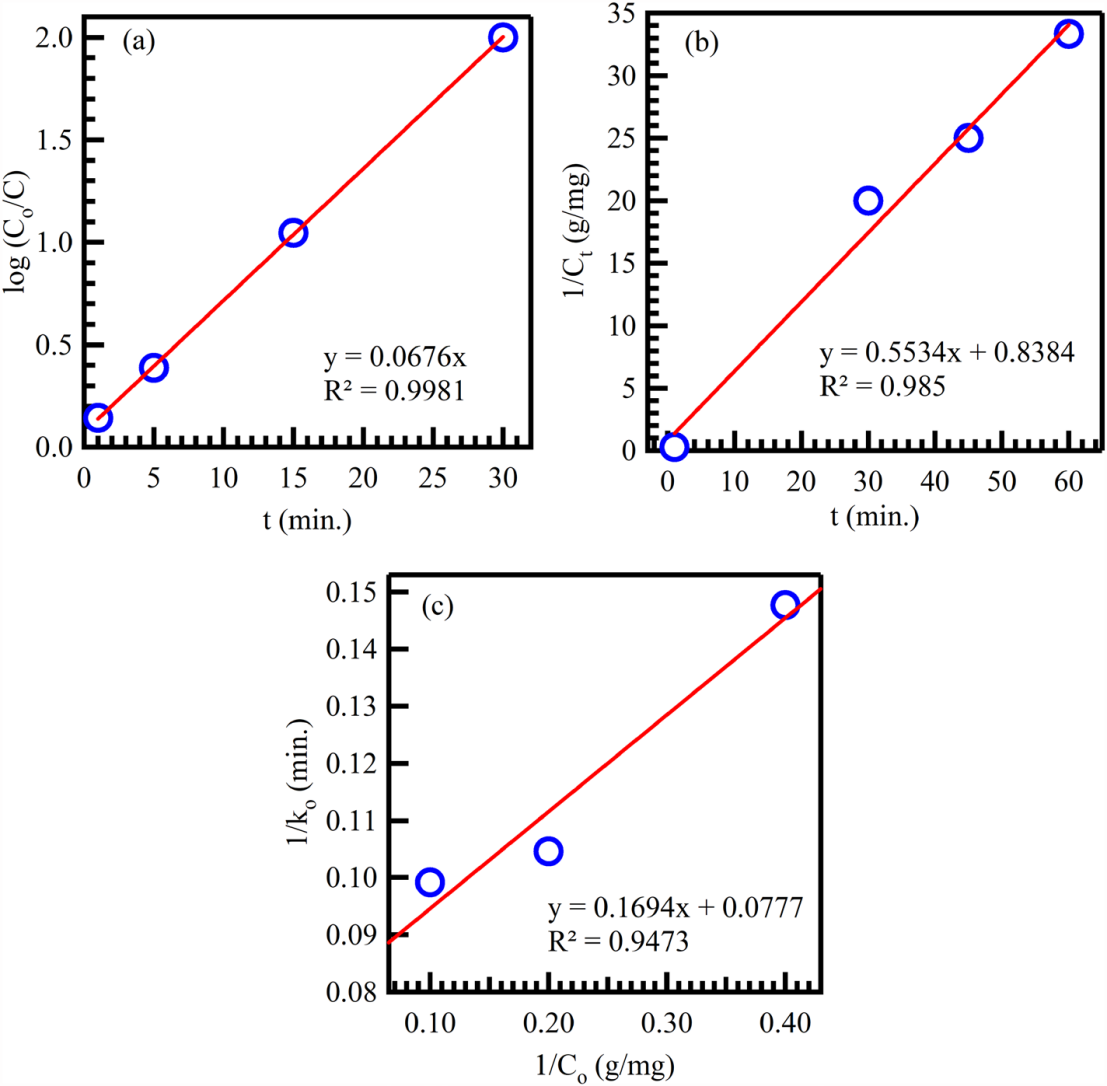
Pseudo-first-order (**a**), pseudo-second-order (**b**), Langmuir–Hinshelwood (**c**) kinetic models for photocatalytic degradation of CV using Au@rGO nanocomposite.

In these equations, C_o_ represents the initial CV concentration, C_t_ represents the final CV concentration after a certain period, k_1_ is the rate constant for the pseudo-first-order model, and k_2_ is the rate constant for the PSO model. Figure 12 illustrates the kinetic study of CV photocatalytic degradation on the surface of the Au@rGO nanocomposite. The plot shows ln(C_o_/C_t_) and 1/C_t_ on the Y-axis against irradiation time on the X-axis, representing the PFO and PSO kinetics of CV-photocatalytic degradation on the Au@rGO nanocomposite, respectively (Fig. 12a,b).

Additionally, Table 3 summarizes various kinetic parameters such as the rate constants k1 and k_2_ for the PFO and PSO models, respectively, the half-life period (t_0.5_), and the coefficient of determination (R^2^). The half-life time of the reaction, t_0.5_ (in minutes), can be calculated using the formulas (0.693/k1) for PFO and (1/k_2_C_o_) for PSO. The kinetic data presented in the table represent the CV photocatalytic degradation kinetics study of Au@rGO nanocomposites. The R^2^ values for PFO and PSO are 0.9968 and 0.9850, respectively, indicating that the data for PFO fits the model better than PSO. Therefore, to achieve complete CV removal through the photocatalytic degradation approach, CV molecules must be adsorbed on the surface of the Au@rGO nanocomposite.

The rate of CV degradation using Au@rGO nanocomposite can be evaluated through the application of Langmuir–Hinshelwood using the following equation^83^:

18 $$\frac{1}{{k_{o} }} = \frac{1}{{k_{r} }} + \frac{1}{{k_{r} k_{s} C_{o} }}$$

In Eq. (10) k_o_ is the initial rate, C_o_ is the initial concentration of CV, k_r_ is the apparent kinetic constant and k_s_ is the adsorption constant. The first-order rate constant values were further utilized to explore the Langmuir–Hinshelwood model for CV removal using Au@rGO nanocomposite at different initial CV concentrations, as depicted in a graph between 1/k_o_ and 1/C_o_ (Fig. 12c). Values of K_r_ and K_s_ were defined from the slope and intercept of the plot, resulting in K_r_ being 12.87 mg/L.min and K_s_ being 0.459 L/mg. These results illustrate that when K_r_ > K_s_, CV trapping onto the surface of the Au@rGO nanocomposite was the controlling step of the photodegradation process.

### Regeneration and reusing of Au@rGO

**Figure 13:**
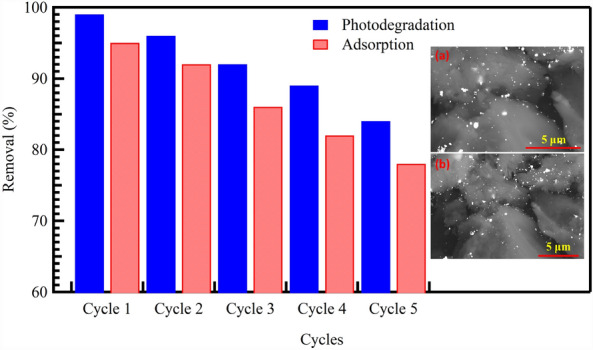
Cycling performance of the Au@rGO, insets show SEM images of Au@rGO nanocomposite before (**a**) and after (**b**) five reusing cycles.

The reusability and repeated use cycles of Au@rGO sorbent/catalysts offer important prospects for laboratory and scale-up applications. Because of their simplicity, safety and the possibility of reuse, the adsorption and photolysis processes are the most cost-effective methods for removing CV dye from wastewater. By employing the elution process, it becomes feasible to reuse the discarded adsorbent/photocatalyst multiple times. This leads to a reduction in the overall expenditure associated with the removal of the adsorption of CV dye at the inner/outer active sites of the surface of the mesoporous Au@rGO adsorbent/catalyst, whether via adsorption or photocatalytic degradation, which can potentially decrease the availability of these active sites. Consequently, this is resulting in a reduction in the efficiency of Au@rGO for the adsorption and photocatalytic degradation of CV dye. Therefore, the goal of these experiments is to efficiently analyze how to utilize the synthesized materials used as adsorbents/catalysts for using them in the long term. Nitric acid had been used as an eluent solution and the influence of HNO_3_ concentration and elution time were performed through batch experiments to investigate the efficiency of CV-elution. The regenerated CV-free Au@rGO solid was filtered, dried, and reused as displayed in Fig. 13, the efficiency of the adsorption and photocatalytic degradation of CV dye using Au@rGO in the first cycle reached about 95% and 99%, respectively. Then its efficiency decreases at cycle number five from the repeated experiments 78% and 84%, respectively. Based on these findings, the reusability of Au@rGO nanocomposite numerous times enabled a low-cost technique for real-world large-scale applications. The SEM micrographs of the Au@rGO nanocomposite before and after five cycles confirmed the high stability of its morphology even after many reusing cycles, as shown in the inset of Fig. 13.

## Conclusions

In this work, Au@rGO nanocomposite was synthesized using sustainable, simple, and cost-effective method via recycling the electronic waste. The nanocomposite showed high adsorption and photocatalytic degradation ability for CV removal from polluted water. At pH 10 and 30 min, the nanocomposite can remove more than 99% of CV content from wastewater under irradiation. The adsorption of CV dye on the Au@rGO nanocomposite follows the Langmuir model, which means that the CV molecules are adsorbed onto the surface of the nanocomposite to form a monolayer. The theoretical value of the CV-adsorbed amount was calculated based on the Langmuir isotherm and it was found to be 55 mg/g, which agrees with the actual value of the CV adsorbed amount. The kinetic data obtained for both adsorption and photocatalytic degradation of CV dye on the surface of the Au@rGO nanocomposite fit better into the PFO kinetic model than the PSO kinetic model. The Au@rGO nanocomposite can be readily regenerated and reused for several cycles with high efficiency of 99%. The recycled Au@rGO nanocomposite is a promising candidate for water purification technologies (CV removal).

## Data Availability

All data generated or analysed during this study are included in this published article.
